# Isolation of *Bartonella* sp. from Sheep Blood

**DOI:** 10.3201/eid1310.070570

**Published:** 2007-10

**Authors:** David A. Bemis, Stephen A. Kania

**Affiliations:** * University of Tennessee, Knoxville, Tennessee, USA

**Keywords:** Bartonella, sheep blood, bacteremia, contaminant, dispatch

## Abstract

A *Bartonella* sp. was isolated from sheep blood. Bacterial identification was conducted by using electron microscopy and DNA sequencing of the 16S rRNA, citrate synthase, riboflavin synthase, and RNAase P genes. To our knowledge, this is the first report of ovine *Bartonella* infection.

*Bartonella* spp. are potential zoonotic pathogens that frequently cause bacteremia without overt disease in reservoir hosts (*1*). Several new *Bartonella* spp. were found in wild and domestic ruminants from Europe and North America (*2*–*4*), but previous blood cultures performed on 150 domestic sheep and 84 bighorn sheep failed to isolate *Bartonella* spp. (*2*,*5*).

## The Study

**Figure 1:**
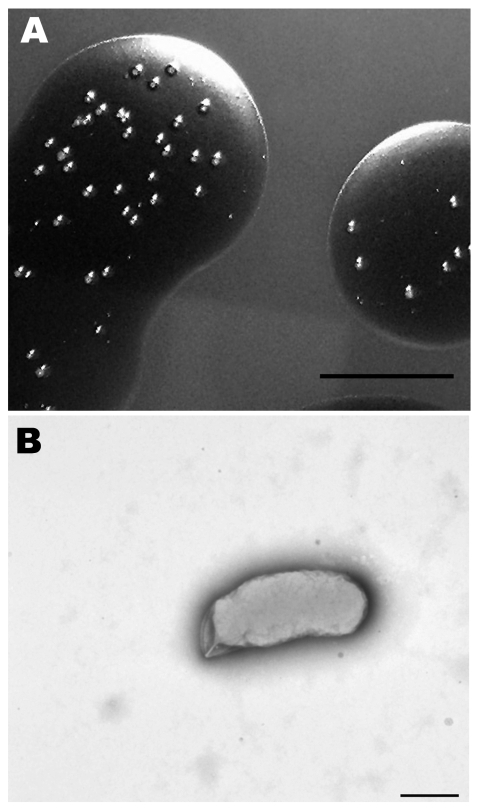
Morphologic analysis of a Bartonella sp. isolated from sheep blood. A) Colonies growing in sheep blood surface biofilm seen in reflected light after 25 days. Scale bar = 10 mm. B) Transmission electron micrograph of a representative cell that was dispersed from a 25-day-old colony and negatively stained with 0.5% potassium phosphotungstic acid. Scale bar = 500 nm

We isolated a *Bartonella* sp. from 2 successive lots of commercial defibrinated sheep blood received from mid- February to mid-March 2007, from a US supplier. Bottles (10 from lot 1 and 6 from lot 2), each containing 100 mL, were newly opened on the day of receipt. Approximately 0.2 mL was aseptically removed and spotted onto the surface of a Columbia agar (BBL; Becton Dickinson, Sparks, MD, USA) plate containing 5% defibrinated sheep blood. The remaining blood was stored at 4°C. Plates were incubated at 35°C in an atmosphere of 7% CO_2_ and examined daily for bacterial growth. Blood appeared sterile after 7 days; however, by 14–21 days, pinpoint bacterial colonies were recognized in the blood film. After 3–4 weeks, mature colonies (Figure 1, panel A) were rough, off-white, difficult to disperse but nonadherent to the agar surface, and ≈1 mm in diameter. Monomorphic colonies grew in all samples within each lot. Estimated concentrations in the starting pools of blood from lots 1 and 2 were 750 CFU/mL and 25 CFU/mL, respectively.

The cells were small, gram-negative rods, 0.47–0.60 μm in diameter, and 0.8–1.9 μm in length (Figure 1, panel B). Flagella were not observed. Growth was not detected after transfer of colonies to Columbia blood agar and several other available diagnostic media that contained blood products (e.g., chocolate agar, Centers for Disease Control and Prevention anaerobe agar, Bordet-Gengou agar, Mycoplasma agar, and hemin-supplemented thioglycolate medium). Colony transfer to Columbia blood agar plates that were first overlaid with sheep blood (from a presumed uninfected lot) resulted in only 2 or 3 colonies. Repeat cultures from lot 1 showed a 97% reduction in colony numbers after 37 days of storage and no growth in samples after 72 days of storage.

**Figure 2:**
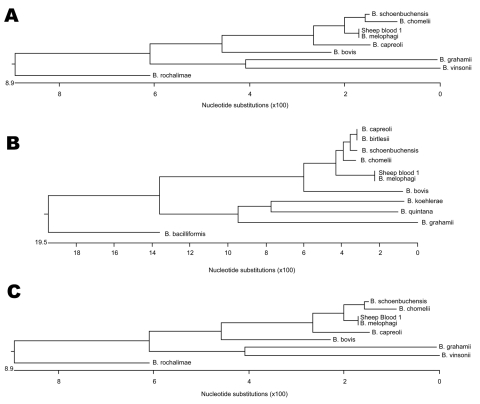
Phylogenetic trees (DNAstar ClustalW Slow and Accurate; DNASTAR Inc., Madison, WI, USA) of 3 Bartonella genes. A) Citrate synthase. B) Riboflavin synthase. C) 16S rDNA. Sheep blood 1 is compared with species showing the highest homology for each gene.

PCR was performed as described (*6*–*8*) by using template DNA obtained from a representative colony from each lot. PCR products from the lot 1 isolate (SB1) and lot 2 isolate (SB 2) had identical DNA sequences (sequencing performed at the University of Tennessee Core DNA Sequencing Facility). Phylogenetic trees of *Bartonella* spp. based on individual 16S rRNA, citrate synthase (*gltC*) and riboflavin synthase (*ribC*) sequence alignments showed greatest similarity to *Bartonella melophagi* (Figure 2).

Initial comparison of the DNA sequence with double-strand agreement from the 16S rRNA gene most closely matched (1,283/1,286 bp [99%]) that of *Wolbachia melophagi* (GenBank accession no. X89110). However, a shorter sequence (1,212 bp) from the SB1 isolate aligned closely (99.8%) with both *W*. *melophagi* and *B*. *melophagi* (GenBank accession nos. X89110 and AY724770). The *gltC* gene sequences were consistent with those of *B*. *melophagi* (GenBank accession nos. AY692475, AY724769, and AY724768). The matches of both DNA strands were 275/275 bp (100%) with each strain. The DNA sequence with double-strand agreement from the *ribC* gene also matched (473/473bp [100%]) that of *B. melophagi* (GenBank accession no. EF605287). The RNAase P gene (*rnpB*) sequence had more distant matching (95.6%) with sequences of *B*. *weissi* (GenBank accession no. AF376050) and *B*. *sp*. Deer 159/660/1 (95.7%) (GenBank accession no. AF376051). DNA sequences from the *B*. *melophagi rnpB* gene were not available in GenBank for comparison. The DNA sequences determined in this study have been assigned the following GenBank accession nos.: 16S rRNA (EF689897), citrate synthase (EU020109), riboflavin synthase (EU020110), and RNAase P (EU020111).

## Conclusions

The source of *Bartonella* sp. was likely intrinsic contamination from bacteremia in donor sheep. Blood was obtained from multiple live sheep with sterile, closed blood collection systems and from venipuncture sites that were prepared by shearing and treatment with antiseptics. Each 5-L lot (1 L/sheep) was pooled and prepared for sale in a separate, clean, well-equipped laboratory facility. Histories of sheep were not determined. Young age (*9*) and contact with wildlife (*2*) or cross-species vectors (*5*) may increase the risk for *Bartonella* infection in sheep.

Arthropod vectors often transmit *Bartonella* infections. *Melophagus ovinus*, commonly called a sheep ked, is a hemophagous ectoparasite of sheep (*5*). The organism from which DNA sequence of a 16S rRNA gene was isolated was an uncultured bacterial endosymbiont of sheep keds initially called *W*. *melophagi*. However, taxonomists now agree that the organism from which the original sequence came should be removed from the genus *Wolbachia* and placed in the genus *Bartonella* (*5*). On the basis of DNA sequence data, candidate status was proposed for the new species *B*. *melophagi* (M. Vayssier-Taussat, L. Halos, H.-J. Bouluis, unpub. data, available from www.ncbi.nlm.gov/taxonomy/browser/wwwtaxcgi?id = 291176). An organism with DNA sequence matching that of *B*. *melophagi* was recently isolated from a sheep ked (M.Y. Kosoy, K.W. Sheff, A.I. Irkhin, unpub. data, available from www.ncbi.nlm.gov/taxonomy/browser/wwwtax.cgi?id = 291176).

Sheep blood is often used in the laboratory with the expectation that it is free of bacteria. However, routine animal health surveillance and quality control procedures may fail to detect *Bartonella* spp. Optimal growth conditions for this organism are unknown. In this study, growth was only observed in fresh sheep blood. We were unable to obtain sufficient growth after in vitro passage for further phenotypic characterization. A novel liquid culture medium that supported growth of *Bartonella* spp. also used fresh, defibrinated sheep blood as a growth supplement (*10*). High-level *Bartonella* bacteremia may be transient, and the sensitivity of PCR for detection in pooled blood or individual sheep has not been established. PCR assays performed on lots 1 and 2 after 1 month of storage did not detect *Bartonella* spp. PCR was not performed at the time of blood collection. Risks associated with *Bartonella* infection in sheep are unknown. Precautions to reduce potential transmission of *Bartonella* are advised when handling sheep blood.
